# HP1330 Contributes to *Streptococcus suis* Virulence by Inducing Toll-Like Receptor 2- and ERK1/2-Dependent Pro-inflammatory Responses and Influencing *In Vivo S. suis* Loads

**DOI:** 10.3389/fimmu.2017.00869

**Published:** 2017-07-31

**Authors:** Qiang Zhang, Jingjing Huang, Junping Yu, Zhongmin Xu, Liang Liu, Yajing Song, Xiaomei Sun, Anding Zhang, Meilin Jin

**Affiliations:** ^1^National Key Laboratory of Agricultural Microbiology, Huazhong Agricultural University, Wuhan, China; ^2^College of Veterinary Medicine, Huazhong Agricultural University, Wuhan, China; ^3^Key Laboratory of Development of Veterinary Diagnostic Products, Ministry of Agriculture, Wuhan, China

**Keywords:** *Streptococcus suis* 2, streptococcal toxic shock-like syndrome, excessive inflammation, signaling pathway, recognition receptor

## Abstract

*Streptococcus suis* 2 (SS2) has evolved into a highly invasive pathogen responsible for two large-scale outbreaks of streptococcal toxic shock-like syndrome (STSLS) in China. Excessive inflammation stimulated by SS2 is considered a hallmark of STSLS, even it also plays important roles in other clinical symptoms of SS2-related disease, including meningitis, septicemia, and sudden death. However, the mechanism of SS2-caused excessive inflammation remains poorly understood. Here, a novel pro-inflammatory protein was identified (HP1330), which could induce robust expression of pro-inflammatory cytokines (TNF-α, MCP-1, and IL-1β) in RAW264.7 macrophages. To evaluate the role of HP1330 in SS2 virulence, an *hp1330-*deletion mutant (*Δhp1330*) was constructed. *In vitro, hp1330* disruption led to a decreased pro-inflammatory ability of SS2 in RAW 264.7 macrophages. *In vivo, Δhp1330* showed reduced lethality, pro-inflammatory activity, and bacterial loads in mice. To further elucidate the mechanism of HP1330-induced pro-inflammatory cytokine production, antibody blocking and gene-deletion experiments with macrophages were performed. The results revealed that the pro-inflammatory activity of HP1330 depended on the recognition of toll-like receptor 2 (TLR2). Furthermore, a specific inhibitor of the extracellular signal-regulated kinase 1/2 (ERK1/2) pathways could significantly decrease HP1330-induced pro-inflammatory cytokine production, and western blot analysis showed that HP1330 could induce activation of the ERK1/2 pathway. Taken together, our findings demonstrate that HP1330 contributes to SS2 virulence by inducing TLR2- and ERK1/2-dependent pro-inflammatory cytokine production and influencing *in vivo* bacterial loads, implying that HP1330 may be associated with STSLS caused by SS2.

## Introduction

“*Streptococcus suis* is responsible for severe economic losses in the worldwide swine industry and poses serious threats to human health” (1). In general, “of the 29 described serotypes, serotype 2 (SS2) is the most prevalent in humans” (2), but human infections with other serotypes also occur sporadically (3). Since “the first human case was reported in Denmark in 1968” (4), to date, >1,500 *S. suis* infections in humans have been documented worldwide (5). Although most reports concerned sporadic cases of infection, two recent large-scale outbreaks of human SS2 occurred in China (6, 7). In addition, “a large series of 151 *S. suis* meningitis cases was also reported in southern Vietnam” (8). “SS2 has evolved into a severe pathogen, particularly in light of patients presenting with streptococcal toxic shock-like syndrome (STSLS), indicating that new, highly virulent bacterial variants have emerged recently in Asia” (9).

In previous studies, several virulence-related factors of SS2 were identified, such as capsular polysaccharide, muramidase-released protein, suilysin, subtilisin-like protease, and IgA1 protease (10–12). However, current knowledge regarding the pathogenesis of SS2 infection remains limited, particularly for STSLS (13). In general, streptococcal toxic-shock syndrome (STSS) is toxin-mediated and associated primarily with superantigens. However, no putative superantigen or homologous gene was identified in the genomes of SS2 isolates associated with STSLS, indicating that several unique mechanisms could be involved (14). Excessive inflammation, as a hallmark of SS2 infection, is responsible for most clinical signs of SS2-related pathology leading to meningitis, septicemia, STSLS, and sudden death (7, 15–17). Therefore, explaining the mechanisms of excessive inflammatory responses induced by SS2 could help understand the pathogenesis, even of STSLS caused by SS2. As a Gram-positive bacterium, SS2 produces some common pathogen-associated molecular pattern (PAMP) molecules, including peptidoglycan (PGN), lipoteichoic acid, and lipoproteins, which can induce the release of cytokines and chemokines (18). Indeed, several previous studies have shown that PGN, LTA, and some lipoproteins are associated with SS2 virulence (19–22). However, little evidence indicates that these PAMPs are responsible for excessive inflammatory responses, even STSLS caused by SS2. At present, the mechanism whereby SS2 causes excessive inflammation remains poorly understood.

To explore the mechanisms of excessive inflammation stimulated by SS2, we investigated novel pro-inflammatory mediators of SS2. In our previous study, over 50 extracellular SS2 proteins were expressed in *Escherichia coli* and purified using a His-tag (18), and these proteins had been previously described as secreted proteins, cell wall proteins, and membrane proteins (23–25). Several novel pro-inflammatory proteins were identified (data not shown), of which HP1330 (encoded by SSUSC84_1330) displayed rather potent pro-inflammatory activity. In present study, we sought to evaluate the role of HP1330 in SS2 infection and elucidate the mechanism through which it induces pro-inflammatory responses.

## Materials and Methods

### Bacterial Strains, Plasmids, and Growth Conditions

In Table 1, we showed the information of bacterial strains and plasmids used in this study. SS2 strain SC19 was selected as the wild-type (WT) strain, which “was isolated from the brain of a dead pig during the epidemic outbreak in the Sichuan Province of China in 2005” (26). SC19 is highly pathogenic to mice and pigs and can cause STSLS (27). SC19, *Δhp1330*, and *CΔhp1330* were cultured in tryptic soy broth (TSB) or on tryptic soy agar (TSA) plates (Difco, MI, USA) with 10% newborn bovine serum (Sijiqing Biological Engineering Materials Co., Ltd., Hangzhou, China) at 37°C (28).

**Table 1:** Summary of bacterial strains and plasmid used in this study.

Group	Names	Characteristics and functions	Sources or references
Bacterial strains	SC19	*Streptococcus suis* serotype 2, wide type	(27)
	*Δhp1330*	*hp1330-*deletion mutant strain	This study
	*CΔhp1330*	complemented strain of *hp1330*	This study
	*Escherichia coli* DH5α	Cloning host for recombinant vector	Trans
	*E. coli* BL21	Expression host for recombinant protein	Trans
Plasmids	pET28a	Expression vector; Kan^r^	Novagen
	pSET4s	*E. coli*–*S. suis* shuttle vector; Spc^r^	(29)
	pSET2	*E. coli*–*S. suis* shuttle vector; Spc^r^	(29)
	p4*Δhp1330*	Derived from pSET4s used to knock out *hp1330* in SC19; Spc^r^	This study
	p2*CΔhp1330*	Derived from pSET4s used to complement *hp1330* in *Δhp1330*; Spc^r^	This study

*Kan^r^, kanamycin resistant; Spc^r^, spectinomycin resistant*.

### Preparation of the Recombinant HP1330 Protein

The HP1330 protein was prepared according to published methods (30). Briefly, the *hp1330* gene was amplified by PCR using the primers listed in Table 2, and then inserted into the pET28a vector. After the recombinant vector was transformed into *E. coli* BL21 (DE3) cells, 0.5 mM isopropyl-b-d-thiogalactopyranoside was added to induce expression. Then HP1330 was purified by ultrasonication and Ni-NTA agarose chromatography. Before being used to stimulate RAW264.7 macrophages, HP1330 was confirmed to contain low levels of endotoxin, using the Endotoxin Removal Kit (Genmed Scientifics Inc., USA) and Quantitative Chromogenic Tachypleus Amebocyte Lysate for Endotoxin Detection Kit (Chinese Horseshoe Crab Reagent Manufactory Co., Ltd., Xiamen, China) (31). After passage through a 0.22-µm filter, the HP1330 protein was stored at −80°C.

**Table 2:** Oligonucleotide primers used in this study.

Primers	Primers sequence (5′–3′)^a^	Functions
*hp1330*-F	CGCGAATTCGAAAGCAATACTGCGACTGT	For amplification of the *hp1330* ORF gene
*hp1330*-R	CGCCTCGAGCTATTCTGAATACAAGGCAAGG	
*hp1330*L-1	AAAGAATTCGCACGGTATGGGAGGA	Upstream border of *hp1330*
*hp1330*L-2	CCGGATCCAGACTATACCTCTTTCTAGAAATAGG	
*hp1330*R-1	CCGGATCCAAGGGAAAATATGCTTCG	Downstream border *hp1330*
*hp1330*R-2	CCAAGCTTAGGTAAGAAAGGGACAAATC	
c*hp1330*-1	CGCGCATGCGTTAGAAATTGCTAAACAATCCG	For PCR to complement *hp1330*
c*hp1330*-2	CGCGAATTCCTATTCTGAATACAAGGCAAGG	
MCP1-F	AGAAGGAATGGGTCCAGACATA	For qRT-PCR assay
MCP1-R	GTGCTTGAGGTGGTTGTGGA	
TNFα-F	GAGTGACAAGCCTGTAGCCC	For qRT-PCR assay
TNFα-R	GACAAGGTACAACCCATCGG	
IL1β-F	TCATTGTGGCTGTGGAGAAGC	For qRT-PCR assay
IL1β-R	TCATCTCGGAGCCTGTAGTGC	
GAPDH-F	TGGCCTTCCGTGTTCCTAC	For qRT-PCR assay
GAPDH-R	TGAAGTCGCAGGAGACAACC	
P1	CGTCGTATCTGAACCATTG	For PCR to detect the pSET4s
P2	TGGAGAAGATTCAGCCACT	
P3	TGGAAATGTTCAAGTCAACC	For PCR to detect the *gdh*
P4	CGTTTTTCTTTGATGTCCAC	
P5	GGTGTTATTGGCTTGTGG	For PCR to detect the *hp1330*
P6	GTCGCAGTATTGCTTTCC	

*^a^Underlined nucleotides denote enzyme restriction sites*.

### Cell Culture

“RAW 264.7 macrophages were cultured in Dulbecco’s modified Eagle’s medium supplemented with 10% fetal bovine serum (Gibco, USA) at 37°C in a 5% CO_2_ atmosphere” (32). “Primary mouse macrophages were prepared as described previously” (18). Toll-like receptor 2 (TLR2)-deficient, TLR4-deficient, and WT mice were injected intraperitoneally (i.p.) with 4% thioglycolate (TLR2-deficient and TLR4-deficient mice were obtained from the Collaborative Innovation Center of Model Animal, Wuhan University). Peritoneal exudate cells were harvested 4 days later and identified by microscopic analysis and non-specific esterase staining (33). When >90% of the exudate cells were identified as macrophages, the cells were plated at a density of 10^6^ cells per well in 12-well plates.

### Stimulation of RAW 264.7 Cells with HP1330

RAW 264.7 cells were exposed to 10 µg⋅ml^−1^ HP1330 protein, lipopolysaccharide (LPS, 100 ng⋅ml^−1^, Sigma), and LPS inhibitor polymyxin B (Poly.B, 10 µg⋅ml^−1^, Sigma) for 6 h, as described (34).

### RNA Extraction and qRT-PCR

After RAW 264.7 cells were stimulated, the expressions of TNF-α, MCP-1, and IL-1β were measured by qRT-PCR as reported previously (35). Briefly, the total RNA of cells was extracted using the TRIzol^®^ reagent (Invitrogen, Paisley, UK). Then, complementary DNA was synthesized from 4 µg of the total RNA by using AMV reverse transcriptase (Takara, Japan), as previously described (36). qRT-PCR was performed using ViiA™ 7 Software (Applied Biosystems) with SYBR green PCR Kit (Roche). All of the primers used in qRT-PCR were listed in Table 2. The relative amounts of target gene expression were normalized with GAPDH housekeeping gene, using 2^−ΔΔCt^ method (37).

### Enzyme-Linked Immunosorbent Assays (ELISAs) for Cytokines

“The concentrations of TNF-α, MCP-1, and IL-1β in the cell culture supernatants or serums were determined using commercially available ELISA kits (BioLegend), following the manufacturer’s instructions” (18).

### Knockout and Complement of *hp1330*

The *Δhp1330* mutant strain was constructed as previously described (29). The left (714 bp) and right (688 bp) DNA fragments of *hp1330* were prepared from the SC19 genome using PCR with the primers *hp1330*L-1/2 and *hp1330*R-1/2, respectively. The products were inserted into the pSET4s vector to generate plasmid p4*Δhp1330*. Next, the recombinant vector was electrotransformed into SC19 competent cells. The mutant strain was screened based on spectinomycin resistance and thermosensitive suicide of the pSET4s vector. The suspected mutant was verified using three pairs of primers: P1/P2 (to detect the pSET4s vector), P3/P4 (to detect *gdh*), and P5/P6 (to detect *hp1330*).

The complemented strain of *hp1330* was obtained according to a previous procedure (27). A DNA fragment covering the *hp1330* ORF region and its promoter region was prepared by PCR using the primers *chp1330*-1 and *chp1330*-2. This fragment was then cloned into pSET2 to generate plasmid p2*CΔhp1330*. To obtain the complemented strain *CΔhp1330*, the recombinant plasmid was electrotransformed into *Δhp1330*.

### Experimental Infections *In Vitro* and *In Vivo*

*In vitro*, the WT (SC19), *Δhp1330*, and *CΔhp1330* strains were used to infect RAW 264.7 cells at a dose of 5 × 10^6^ colony-forming units (CFUs). After 6 h, culture supernatants and RAW 264.7 cells were collected for ELISA and qRT-PCR analysis, respectively.

This study was carried out in accordance with the recommendations of the Guide for the Care and Use of Laboratory Animals Monitoring Committee of Hubei Province, China, and the protocol was approved by the Committee on the Ethics of Animal Experiments at the College of Veterinary Medicine, Huazhong Agricultural University. For virulence studies, 6-week-old female C57BL/6 mice (10 mice/group) were challenged i.p. with 6 × 10^8^ CFUs of the SC19, *Δhp1330*, or *CΔhp1330* strain. The infected mice were monitored for clinical signs and survival times for 7 days. In addition, another batch of 60 6-week-old female C57BL/6 mice was randomly assigned to three groups with 20 mice/group and challenged i.p. with a non-lethal dose (2 × 10^8^ CFUs per mouse) of the SC19, *Δhp1330*, or *CΔhp1330* strain. At 3, 6, 9, and 12 h postinfection, an equal number of mice in each group were sacrificed to collect blood, which was used for bacteria counts and ELISAs to measure TNF-α, MCP-1, and IL-1β production (26, 38).

### Investigating the Recognition Receptor of HP1330

It is reported that “TLR2 is the major immune receptor involved in *S. suis* recognition” (39, 40). To investigate which receptor was specifically responsible for HP1330-mediated cytokine upregulation, we first detected TLR2 changes after HP1330 stimulation by qRT-PCR, with TLR4 as a control. Second, antibody blocking assays were performed as previously described (41). After pretreatment with 8 µg of an anti-TLR2 (BioLegend) or anti-TLR4 (BioLegend) antibody for 30 min, RAW264.7 cells were incubated with 10 µg⋅ml^−1^ HP1330 for 6 h. The concentrations of TNF-α, MCP-1, and IL-1β in the culture supernatants were determined by ELISA. On the basis of these experiments, we identified the recognition receptor of HP1330. Finally, TLR2−/− and TLR4−/− macrophages were isolated from TLR2−/− and TLR4−/− mice to verify the above results.

### Analysis of HP1330-Induced Cellular Signal-Transduction Pathways

For cell-signaling analysis, RAW 264.7 cells were incubated with the following specific inhibitors 30 min prior to the addition of HP1330, including SB203580 (for p38 MAPK, 10 µM; Cayman Chemical), SP600125 (for JNK, 10 µM; Cayman Chemical), pyrrolidine dithiocarbamate (PDTC; for NF-κB, 20 µM; Sigma), LY294002 (for PI3K, 20 µM; Cayman Chemical), and U0126 (for ERK1/2, 10 µM; Cayman Chemical) (42). After HP1330 stimulation for 6 h, culture supernatants were collected for ELISAs to measure TNF-α, MCP-1, and IL-1β production. According to the conditions used for cytokine activation, we screened for the signal-transduction molecule induced by HP1330.

To verify the above analysis results, the phosphorylation of HP1330-induced signal-transduction molecules was confirmed by western blotting (43). Briefly, after stimulation with 10 µg⋅ml^−1^ HP1330 for 6 h, RAW 264.7 cells were washed once with cold PBS and incubated on ice for 15 min using radioimmunoprecipitation assay lysis buffer with phosphatase inhibitors (Roche). The supernatants were collected, and their protein concentrations were quantified by Bradford protein assay. Then 40 µg proteins were resolved on a 12% sodium dodecyl sulfate-polyacrylamide gel electrophoresis (SDS-PAGE) gel, followed by electrotransfer to a 0.22-µm nitrocellulose membrane. Activation of ERK1/2 was assessed using a specific antibody against phosphorylated ERK1/2 (Cell Signaling Technology). In addition, β-actin was detected by an anti-β-actin antibody (Wuhan PMK Biotechnology Co., Ltd.), as an internal control. Protein bands were visualized by incubation with a horseradish peroxidase-conjugated secondary antibody and then detected using the ECL System (Amersham Life Science, Arlington Heights, IL, USA).

### Statistical Analysis

Statistical analyses were performed by an unpaired Student’s *t*-test. All assays were repeated at least three times, and a *P* value < 0.05 was considered significant. In the figures, * and ** represent *P* values < 0.05 and <0.01, respectively.

## Results

### HP1330-Induced Expression of Pro-inflammatory Cytokines in RAW 264.7 Macrophages

**Figure 1:**
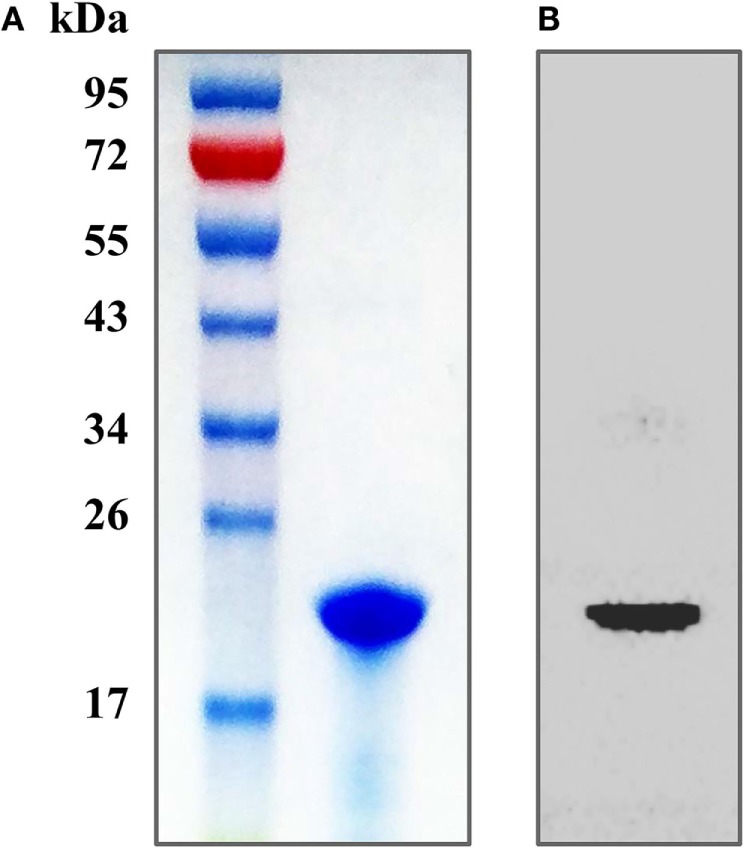
Purification of the recombinant HP1330 protein. **(A)** SDS-PAGE analysis. **(B)** Western blot analysis, the blot was probed with an anti-His tag monoclonal antibody (Cali-Bio).

**Figure 2:**
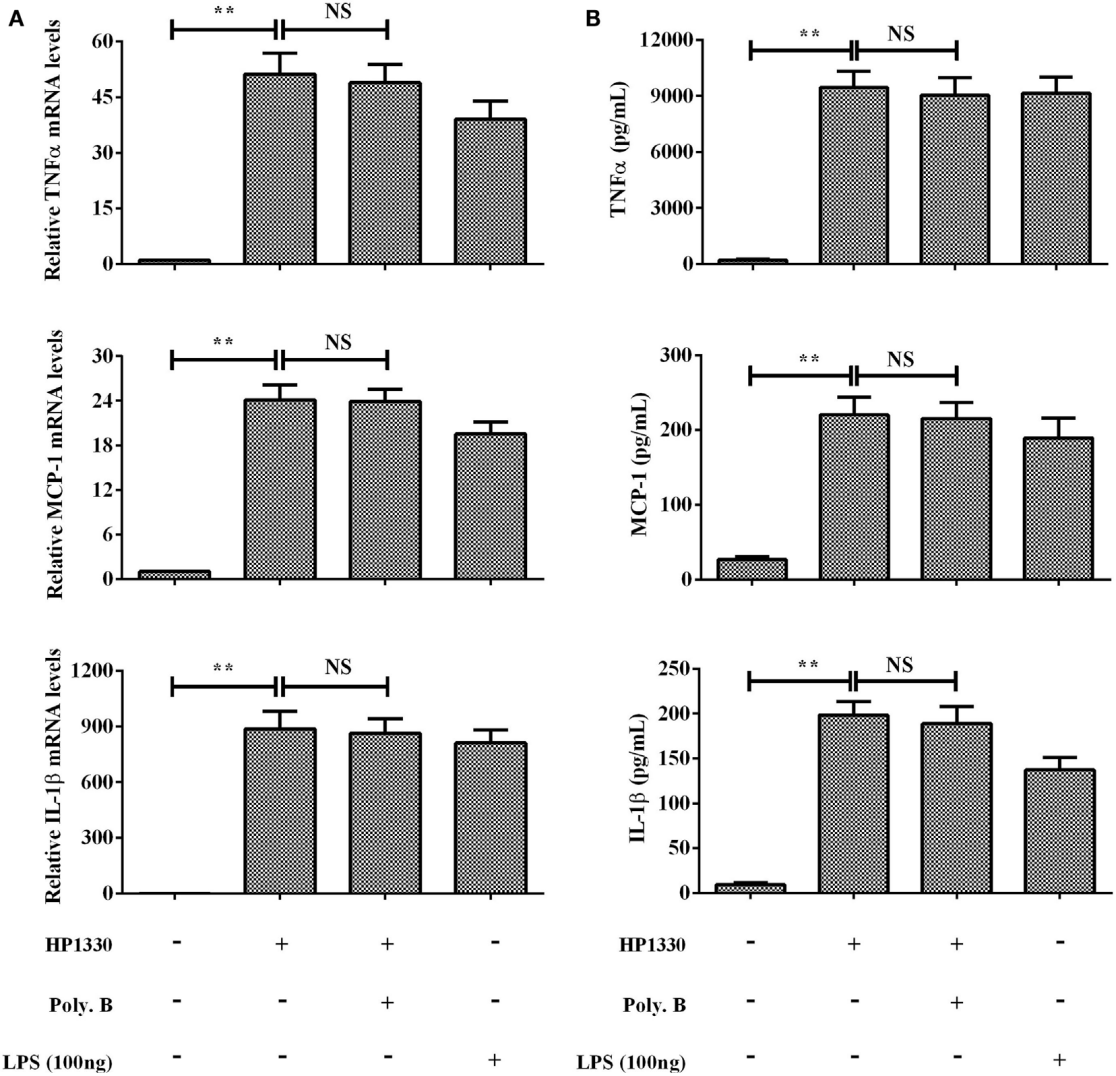
Induction of cytokine mRNA and protein expression in RAW 264.7 macrophages by stimulation with recombinant HP1330. RAW 264.7 macrophages were treated with 100 ng⋅ml^−1^ lipopolysaccharide (LPS) (positive control) or 10 µg⋅ml^−1^ HP1330 protein in the absence or presence of 10 µg⋅ml^−1^ Poly.B for 6 h, or with culture medium (negative control). **(A)** The cytokine mRNA levels were then determined by qRT-PCR, **(B)** and the protein levels of TNF-α, MCP-1, and IL-1β in the culture supernatants were determined by enzyme-linked immunosorbent assay. The bars represent the SEMs, based on three independent experiments. **P < 0.01.

The purity of the recombinant protein HP1330 was analyzed by SDS-PAGE (Figure 1A) and western blotting (Figure 1B), revealing that HP1330 was successfully purified. After endotoxin removal, the protein concentration and endotoxin level of HP1330 were approximately 1.3 mg⋅ml^−1^ and 0.01 endotoxin unit⋅ml^−1^, respectively. Next, we investigated the pro-inflammatory activity of HP1330 in RAW 264.7 cells. As shown in Figure 2, HP1330 significantly induced TNF-α, MCP-1, and IL-1β expression, and the cytokine response induced by HP1330 was not reduced by Poly.B, an inhibitor of negatively charged molecules like LPS that is normally used to exclude the effects of contaminating endotoxins (44, 45). These results showed that HP1330 has robust pro-inflammatory activity in RAW 264.7 cells, and that residual bacterial endotoxins were not responsible for the effect.

### Heat Inactivation of HP1330 Blocked Cytokine Induction

**Figure 3:**
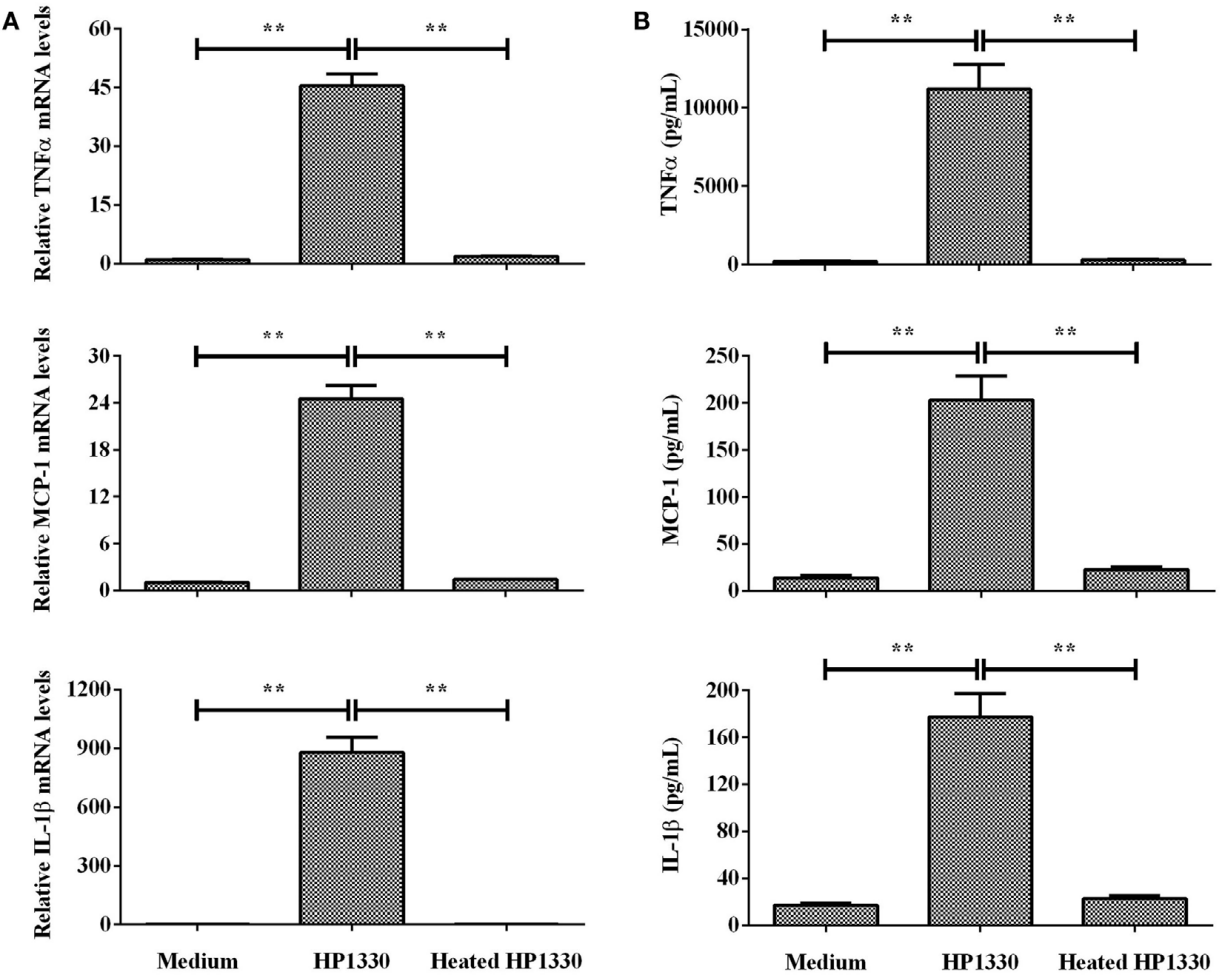
Effect of heat inactivating HP1330 on pro-inflammatory cytokine induction. RAW 264.7 macrophages were stimulated with HP1330 (10 µg⋅ml^−1^) and pretreated HP1330 (10 µg⋅ml^−1^, 100°C for 10 min). **(A)** The mRNA levels of TNF-α, MCP-1, and IL-1β were examined by qRT-PCR, **(B)** and the cytokine protein levels in the culture supernatants were examined by enzyme-linked immunosorbent assay. The bars represent the SEMs, based on three independent experiments. **P < 0.01.

To determine whether heat treatment influences the pro-inflammatory activity of HP1330, we next pretreated HP1330 using previously reported conditions [100°C for 10 min (42)] before adding it to RAW264.7 cells. Heat-treated HP1330 failed to induce TNF-α, MCP-1, and IL-1β production in RAW264.7 cells after a 6-h stimulation (Figure 3). These results indicated that the pro-inflammatory activity of HP1330 in RAW 264.7 cells is heat-sensitive.

### Construction and Characterization of *Δhp1330*

**Figure 4:**
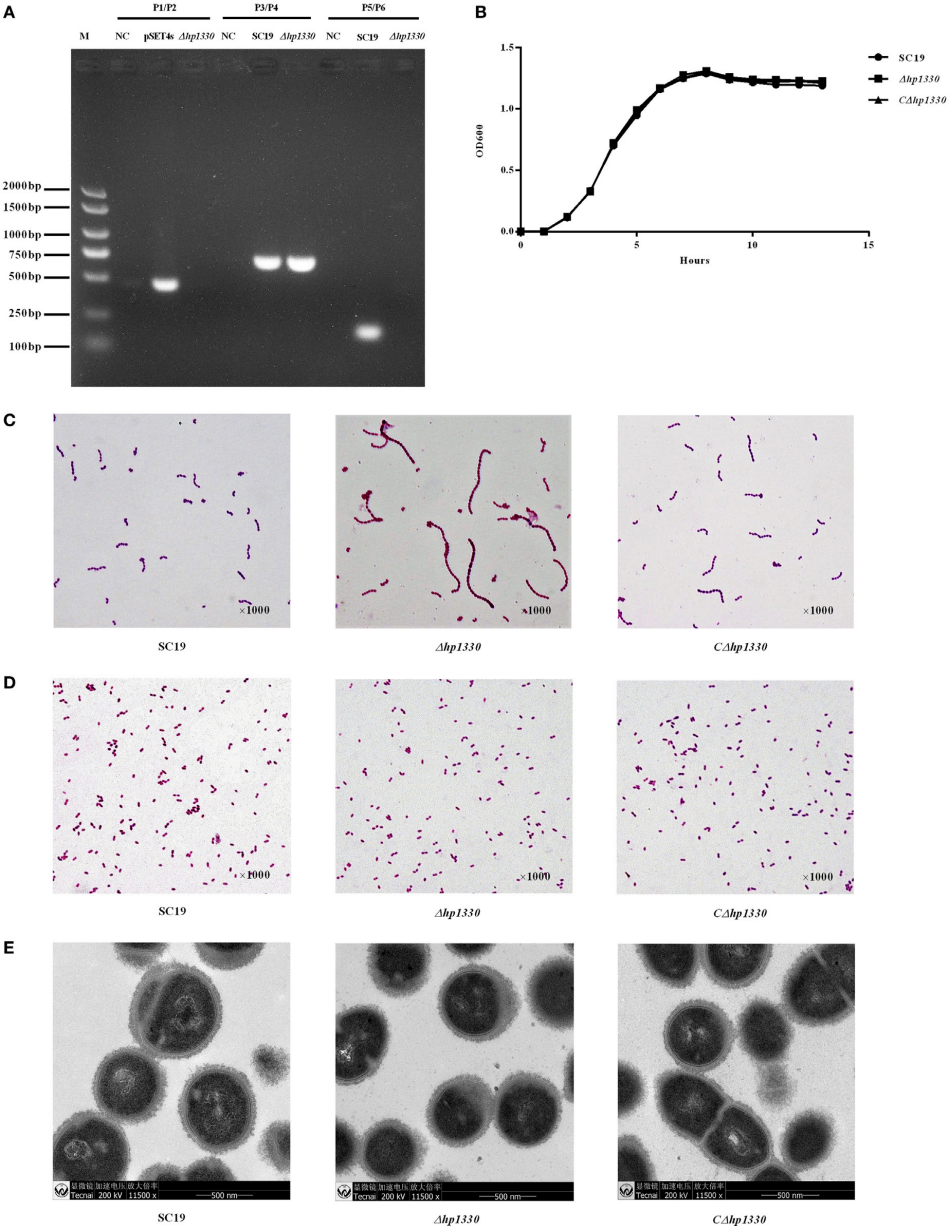
Construction and confirmation of the *Δhp1330* mutant. **(A)** Confirmation of the *Δhp1330* mutant by PCR using the primers pairs P1/P2 (to detect the pSET4s vector), P3/P4 (to detect the *gdh* gene), and P5/P6 (to detect the *hp1330* gene). **(B)** Growth curves of the SC19, *Δhp1330*, and *CΔhp1330* strains. The bacteria were cultured in tryptic soy broth (TSB) containing 5% newborn bovine serum at 37°C. The absorbance at 600 nm was measured at intervals of 1 h. Results shown are representative of three independent experiments. Light microscope morphology of the SC19, *Δhp1330*, and *CΔhp1330* strains were observed by Gram staining (×1,000) following culture in panel **(C)** TSB or in panel **(D)** tryptic soy agar. **(E)** The capsules of SC19, *Δhp1330*, and *CΔhp1330* strains were detected by transmission electron microscopy (×11,500).

To confirm the deletion of *hp1330*, PCR analysis was performed with three pairs of primers (P1/P2, P3/P4, and P5/P6). *hp1330* could be detected in the SC19 strain, but not in *Δhp1330*. As a control, *gdh* could be detected in both SC19 and *Δhp1330*. In addition, the pSET4s vector could not be detected in *Δhp1330* (Figure 4A). These results indicated that the *hp1330* gene was deleted from the bacterial chromosome. To further examine the influence of the *hp1330* deletion, the growth curves and Gram staining of the SC19, *Δhp1330*, and *CΔhp1330* strains were determined by culturing to logarithmic growth phase in TSB. As shown in Figure 4B, no significant difference was found among the growth curves of the three strains, while the chain of *Δhp1330* strain was obvious longer than SC19 or *CΔhp1330* (Figure 4C). To avoid the influence of chain length differences on the accuracy of bacterial counting, we then examined the morphologies of the SC19, *Δhp1330*, and *CΔhp1330* strains cultured on TSA *via* Gram staining. The result showed that no marked difference occurred among these three strains (Figure 4D). Thus, in subsequent cellular and animal experiments, we cultured SC19, *Δhp1330*, and *CΔhp1330* on TSA. In addition, transmission electron microscopy was used to detect the capsules of SC19, *Δhp1330*, and *CΔhp1330* strains, respectively. There was no obvious difference in capsular thickness and morphology among these three strains (Figure 4E). This indicated that HP1330 does not regulate the capsule of *S. suis*.

### *Δhp1330* Inhibited Pro-inflammatory Responses in RAW264.7 Cells

**Figure 5:**
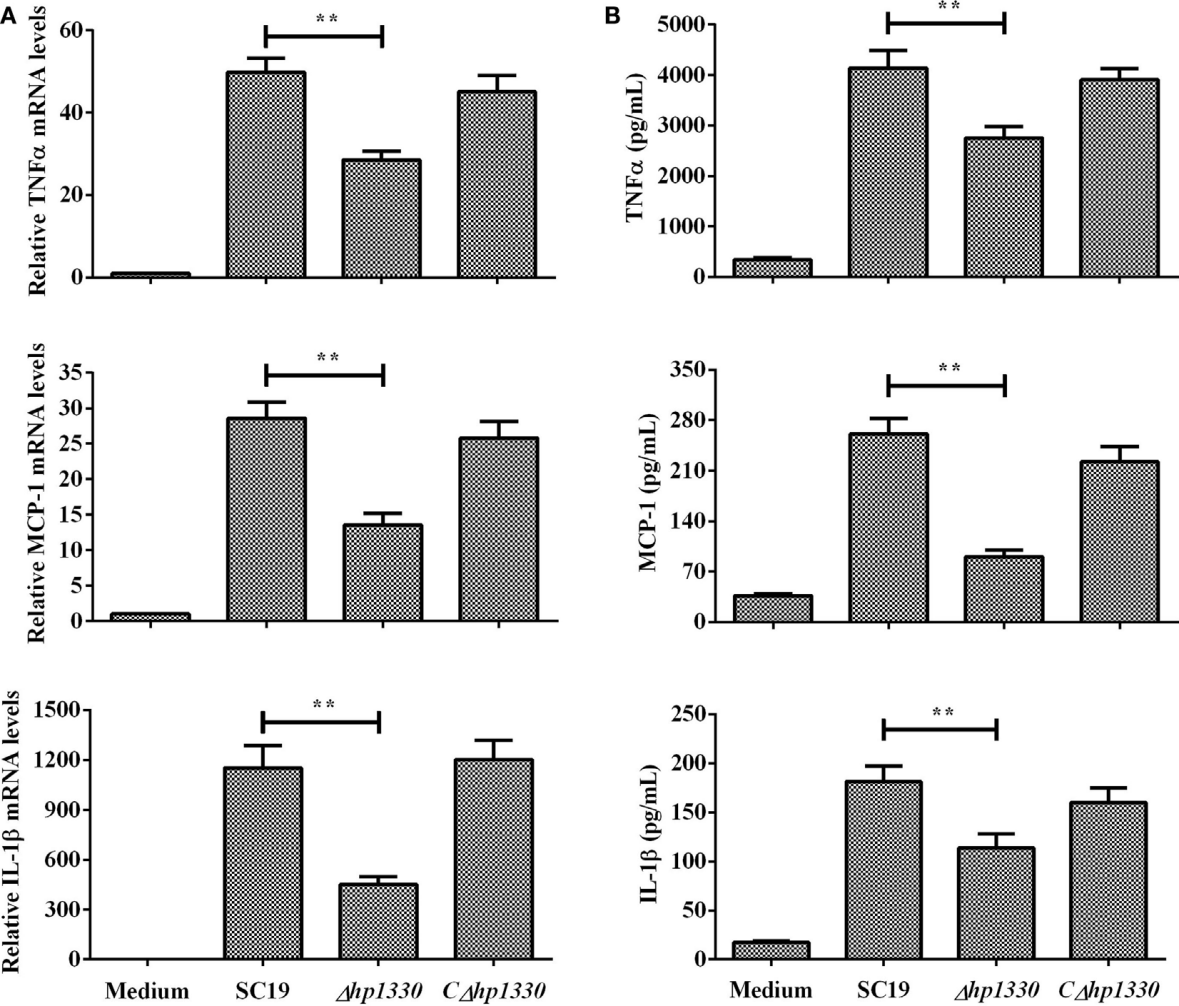
Induction of cytokine mRNA and protein expression in RAW 264.7 macrophages by SS2 strains. RAW 264.7 macrophages were incubated with the SC19, *Δhp1330*, and *CΔhp1330* strains for 6 h. **(A)** The mRNA levels of TNF-α, MCP-1, and IL-1β were examined by qRT-PCR, **(B)** and the cytokine protein levels in the supernatants were examined by enzyme-linked immunosorbent assay. The bars represent the SEMs, based on three independent experiments. **P < 0.01.

After RAW 264.7 cells were incubated with the SC19, *Δhp1330*, or *CΔhp1330* strain, the levels of TNF-α, MCP-1, and IL-1β were measured by ELISA and qRT-PCR. Deleting the *hp1330* gene significantly reduced the pro-inflammatory ability of SS2 (Figure 5). These results indicated that HP1330 plays an important role in SS2-induced, pro-inflammatory responses *in vitro*.

### *Δhp1330* Displayed Attenuated Virulence, Decreased Pro-inflammatory Ability, and Reduced Bacterial Loads in Mice

**Figure 6:**
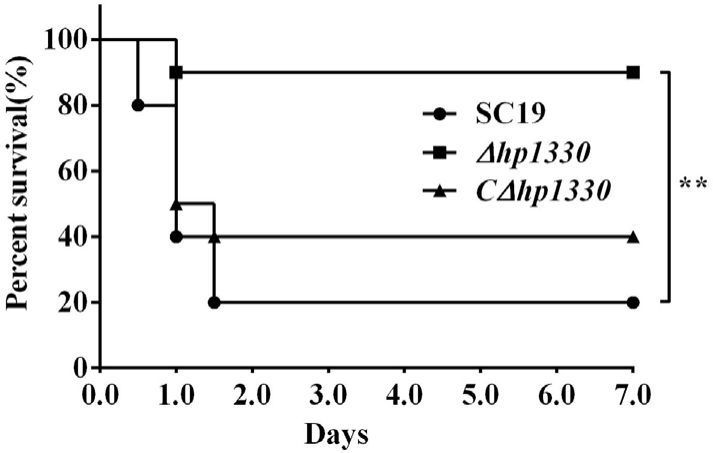
Survival curves of mice infected with SS2 strains. Female C57BL/6 mice in different groups were challenged i.p. with 6 × 10^8^ colony-forming units SC19, *Δhp1330*, or *CΔhp1330* strains cultured on tryptic soy agar. The mortality of mice was recorded for 1 week. The results shown are representative of three independent experiments.

**Figure 7:**
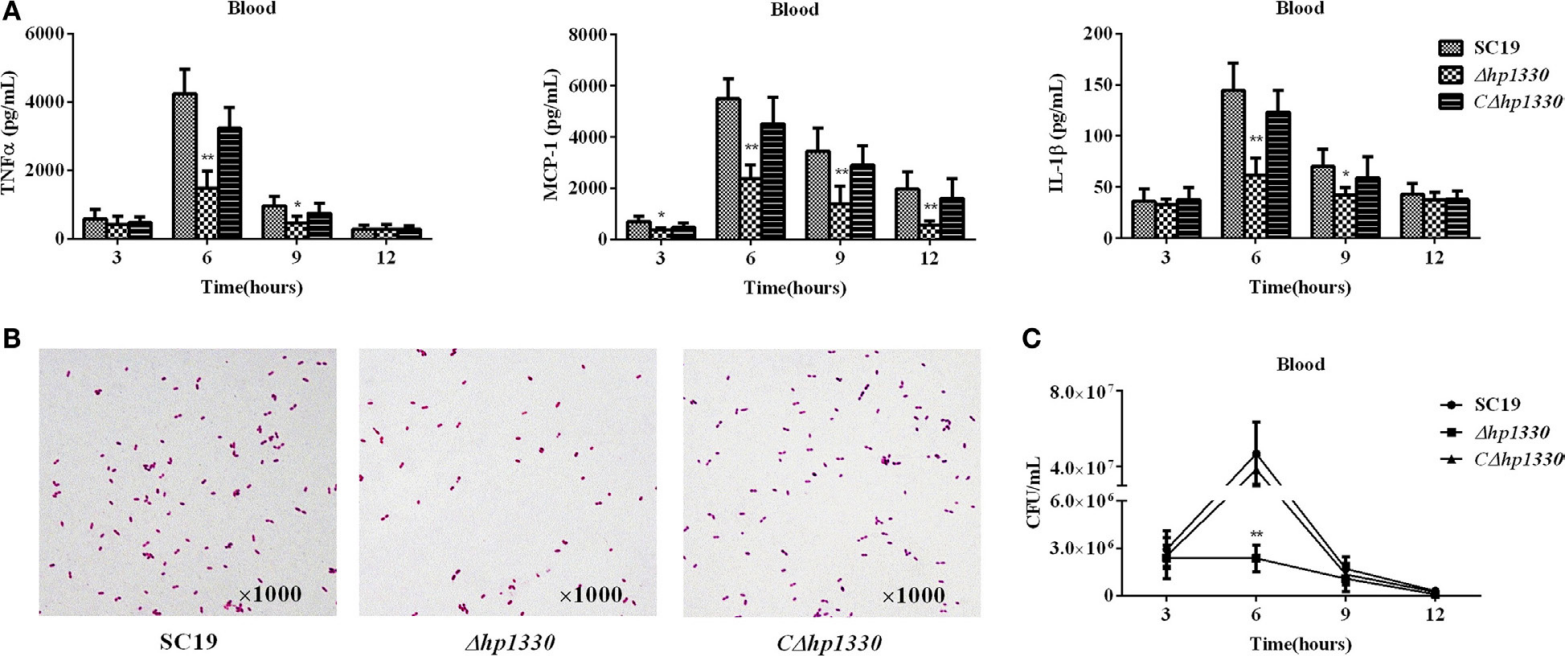
Cytokine concentrations and bacterial loads in blood. Female C57BL/6 mice were challenged with 2 × 10^8^ colony-forming units of the SC19, *Δhp1330*, or *CΔhp1330* strain cultured on tryptic soy agar. After infection for 3, 6, 9, or 12 h, an equal number of mice in each group were sacrificed to collect blood. **(A)** The blood cytokine concentrations were determined by enzyme-linked immunosorbent assay. **(B)** The morphologies of the SC19, *Δhp1330*, and *CΔhp1330* strains from blood were observed under a light microscope by Gram staining (×1,000). **(C)** Bacteria loads in the blood were examined by determining colony-forming unit counts. The results shown are representative of three independent experiments. *P < 0.05, **P < 0.01.

To examine the role of HP1330 in SS2 infection, C57BL/6 mice were used as an experimental infection model. Firstly, for virulence testing, mice were inoculated with the SC19, *Δhp1330*, or *CΔhp1330* strain at a dose of 6 × 10^8^ CFUs, which was a lethal dose of SC19 for C57BL/6 mice. The group of mice infected with SC19 developed obvious clinical signs of SS2 infection, including a rough hair coat, weight loss, depression, shivering, and suppuration of the eyes during the first day postinfection. Only 20% of the mice survived to 7 days postinfection. However, mice in the *Δhp1330* group showed an overall survival rate of 90%, with no obvious symptoms (Figure 6). These results indicated that deleting the *hp1330* gene significantly decreased the virulence of SS2 to mice (*P* < 0.01). Second, to further analyze why the *Δhp1330* deletion resulted in attenuated virulence, mice were challenged i.p. with a non-lethal dose of the SC19, *Δhp1330*, or *CΔhp1330* strain, and then cytokine concentrations and bacterial loads in the blood were determined. As a result, the blood of mice infected with *Δhp1330* contained lower cytokine concentrations at 6 and 12 h postinfection, and lower bacterial loads at 6 h postinfection (Figures 7A,C), whereas the chain length of the SC19, *Δhp1330*, and *CΔhp1330* strains showed no obvious differences (Figure 7B). Overall, our data suggested that HP1330 may contribute to SS2 virulence by inducing high-level pro-inflammatory responses and influencing *in vivo* bacterial loads.

### HP1330-Triggered Pro-inflammatory Cytokine Production Dependent on Recognition of TLR2

**Figure 8:**
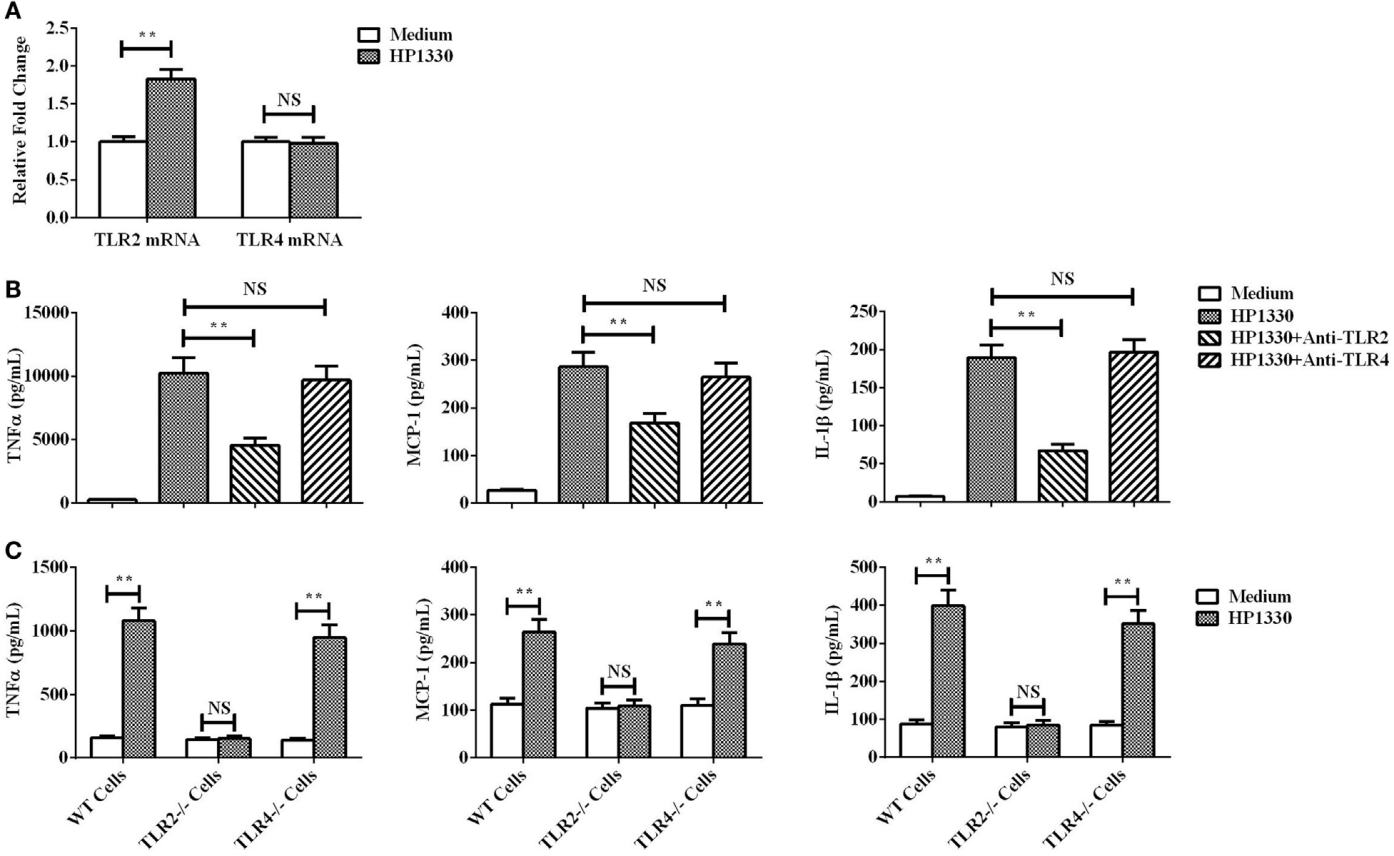
Recognition receptor of the HP1330-stimulated pro-inflammatory response. **(A)** After stimulation with HP1330, RAW264.7 cells were collected to analyze the mRNA levels of toll-like receptor 2 (TLR2) or TLR4 by qRT-PCR. **(B)** Antibody blocking assays. After pretreatment with 8 µg of an anti-TLR2 or anti-TLR4 antibody for 30 min, RAW 264.7 cells were incubated with 10 µg⋅ml^−1^ HP1330 for 6 h. The concentrations of TNF-α, MCP-1, and IL-1β were determined by enzyme-linked immunosorbent assay (ELISA). **(C)** Primary peritoneal macrophages were isolated from TLR2−/−, TLR4−/−, and wild-type (WT) mice, after which they were incubated with 10 µg⋅ml^−1^ HP1330 for 6 h. The cytokine concentrations in the supernatants were determined by ELISA. The error bars represent the SEMs, based on three independent experiments. **P < 0.01.

The qRT-PCR assay results showed that TLR2 could be obviously upregulated in RAW264.7 cells by HP1330 stimulation, but TLR4 not (Figure 8A). This implied that TLR2 may be the inflammatory recognition receptor of HP1330. An antibody blocking assay was performed to test this possibility. Compared with the positive control, the anti-TLR2 antibody significantly reduced the expression levels of TNF-α, MCP-1, and IL-1β (Figure 8B). In addition, TLR2−/− macrophages were isolated from TLR2−/− mice and then used to evaluate the pro-inflammatory activity of HP1330. The results showed that HP1330 could stimulate obvious pro-inflammatory responses in WT macrophages, but not in TLR2−/− macrophages (Figure 8C). All of the above experiments demonstrated that HP1330-induced cytokine secretion depends on TLR2.

### HP1330 Activates Pro-inflammatory Responses Dependent on ERK1/2 Phosphorylation

**Figure 9:**
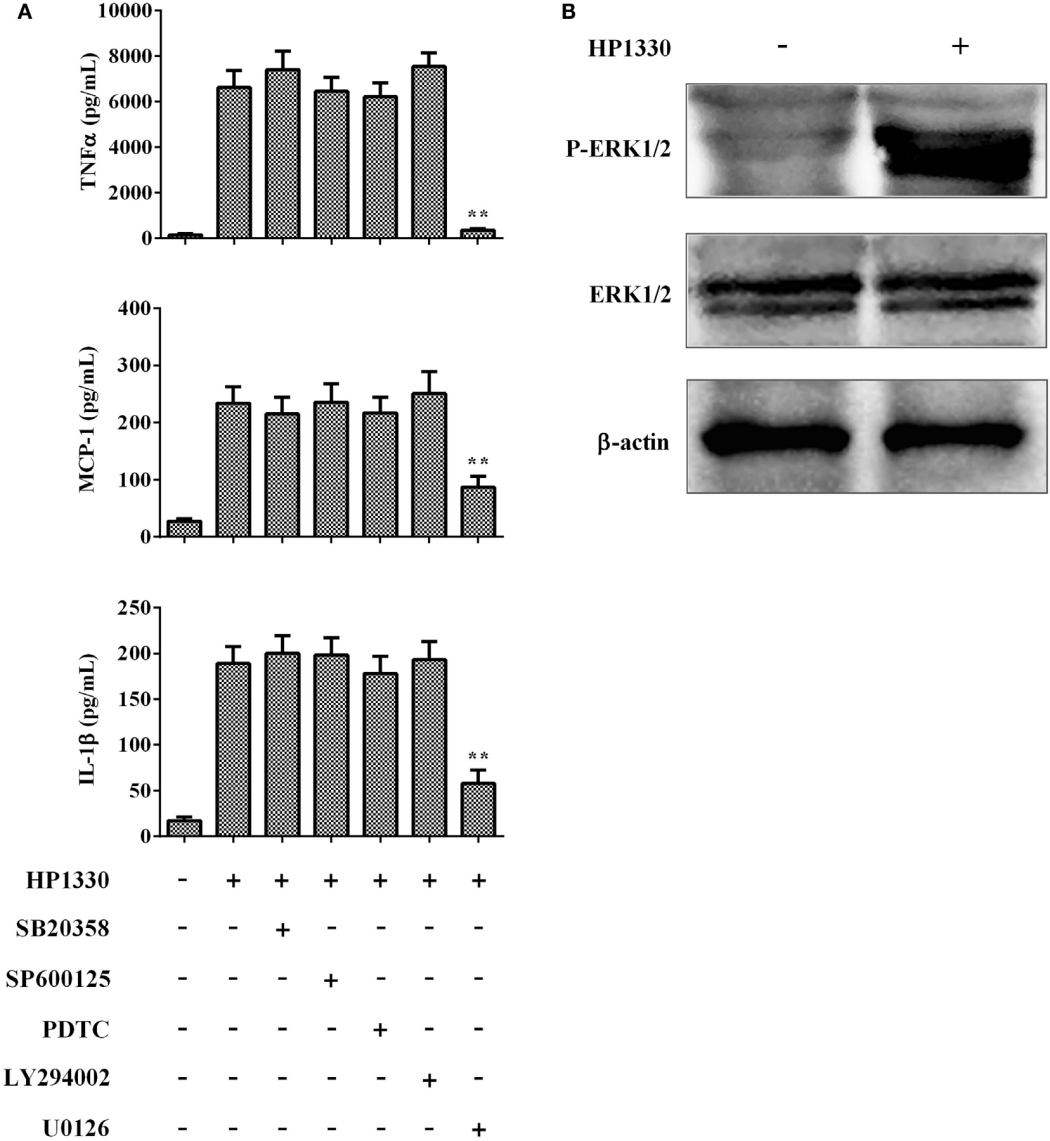
Signal-transduction pathways of the HP1330-stimulated, pro-inflammatory response in RAW 264.7 macrophages. **(A)** After pretreatment with inhibitors of p38 MAPK (SB203580), JNK (SP600125), NF-κB (PDTC), PI3K (LY294002), or ERK1/2 (U0126) for 30 min, RAW 264.7 macrophages were stimulated with 10 µg⋅ml^−1^ HP1330 for 6 h. The cytokine levels were then determined by enzyme-linked immunosorbent assay. Data are expressed as the mean ± SD of three independent experiments. **P < 0.01. **(B)** HP1330-induced phosphorylation of ERK 1/2 MAPK in RAW264.7 macrophages. RAW264.7 macrophages were stimulated with HP1330 (10 µg⋅ml^−1^) for 6 h. The cell lysates were analyzed by western blotting using specific antibodies against ERK 1/2 MAPK and phospho-ERK 1/2 MAPK. β-Actin was detected as an internal control using an anti-β-actin antibody. The results shown are representative of three independent experiments.

To further elucidate the mechanisms through which HP1330-induced pro-inflammatory responses, we investigated HP1330-dependent signal-transduction pathways in RAW264.7 cells. Inhibitors of p38 MAPK, JNK, NF-κB, PI3K, and ERK1/2 were used to analyze which signaling pathway was responsible for HP1330-induced pro-inflammatory responses. As shown in Figure 9A, the ERK 1/2 MAPK inhibitor (U0126) significantly suppressed cytokine production induced by HP1330, whereas the other four inhibitors (SB203580, SP600125, PDTC, and LY294002) did not. This indicated that HP1330-induced pro-inflammatory responses likely depended on ERK 1/2 MAPK phosphorylation. To test this hypothesis, the phosphorylation of ERK 1/2 MAPK was measured by western blot analysis in HP1330-stimulated RAW264.7 cells. Compared with that in the control group, ERK 1/2 MAPK phosphorylation was significantly enhanced in the HP1330-stimulated group, detecting actin as a loading control (Figure 9B). These results indicated that HP1330 activated pro-inflammatory responses in an ERK1/2 phosphorylation-dependent manner.

## Discussion

Currently, *S. suis* remains a major pathogen that causes severe annual economic losses in the global swine industry, and seriously threatens to human health (46). Especially, “two human large-scale outbreaks caused by SS2 in China in 1998 and 2005 have provoked considerable public health concerns worldwide” (12, 47, 48). Although some insights have been gained (49, 50), many aspects of the pathogenesis of the bacteria remain uncertain. For example, the mechanism whereby SS2 causes STSLS still needs to be elucidated. STSLS was first found during the 2005 Sichuan *S. suis* outbreak, with a high (62%) mortality rate (51). Focusing on this novel symptom, some findings indicated that high serum pro-inflammatory cytokine levels and acute bacteremia are responsible for STSLS (7, 52); even several new putative virulence factors were found to be likely associated with STSLS (53–55). However, the exact mechanism whereby SS2 causes STSLS remains unclear. In this study, we found that the *Δhp1330* mutant strain showed clear reductions in lethality, pro-inflammatory ability, and bacterial loads in mice. These results strongly suggested that HP1330 could contribute to SS2-induced STSLS.

When a pathogen invades a host, its PAMP molecules are recognized by the innate immune system of the host *via* pattern-recognition receptors. Subsequently, inflammatory responses are activated to eliminate the pathogen (56). Thus, inflammatory responses are usually beneficial to host (57). However, excessive inflammation is harmful and can lead to shock and organ failure (58). For example, the superantigen secreted by *Streptococcus pyogenes* can induce high levels of inflammatory cytokines and cause STSS (59). Previous findings demonstrated that SS2 has evolved to acquire the ability to stimulate the host immune system to produce massive amounts of pro-inflammatory cytokines, such as TNF-α, IFN-γ, IL-1β, IL-6, IL-12, and MCP-1 (7). Even inflammation has been considered a hallmark of SS2 infection, which plays an important role in most clinical symptoms of *S. suis* disease, including meningitis, septicemia, sudden death, and STSLS (60). Thus, we investigated the mechanisms whereby SS2-induced excessive inflammation contributes to SS2 pathogenesis. In the present study, HP1330 showed potent pro-inflammatory activity *in vitro* and played an important role during SS2-induced excessive pro-inflammatory responses *in vivo*. Through further analyzing the inflammatory signaling pathways activated by HP1330, we found that the pro-inflammatory activity of HP1330 depends on TLR2 recognition and ERK 1/2 MAPK phosphorylation. These results not only demonstrated that HP1330 contributes to SS2 virulence by inducing robust pro-inflammatory responses but they also laid the foundation for attaining a more comprehensive understanding of the excessive inflammation stimulated by SS2.

In this study, we found that when bacteria were cultured in liquid medium (TSB), the chain of *Δhp1330* mutant was longer than WT strain SC19. Because the difference of chain length will influence the accuracy of bacterial counting, to ensure that SC19, *Δhp1330*, and *CΔhp1330* are equally challenged in subsequent cellular and animal experiments, these three strains were cultured on solid medium (TSA), of which the morphologies does not show marked differences. It has been widely reported that PGN hydrolysis is required to promote septal PGN splitting and eventual daughter cell separation (61). Thus, to explore the mechanism whereby HP1330 influences the chain length of SS2, zymogram analysis was performed to analyze PGN hydrolysis induced by HP1330, as described previously (62). Using *S. suis* PGN as the substrate for zymogram analysis, we noticed that HP1330 exhibited apparent enzymatic activity, as did positive control lysozyme, while the negative control protein (bovine serum albumin) did not (Figure S1 in Supplementary Material). These results suggested that HP1330 may influence the chain length of SS2 through PGN hydrolysis. In addition, sequence analysis showed that HP1330 contains a potential zinc-binding site, implying a possible matrix metalloproteinases (MMPs) activity. MMPs contribute to the degradation of the extracellular matrix, which have been extensively recognized in eukaryotes (63). However, the function and mechanism of MMPs in bacteria remain poorly understood, and need further study.

Based on that the deletion of *hp1330* could influence the chain length of SS2 cultured in liquid, in mice experiment, we analyzed the chain length of SC19, *Δhp1330* and *CΔhp1330* strains by Gram staining before bacterial counting. As shown in Figure 7B, no obvious change was observed, excluding the influence of chain length change to bacterial loads. It is known that high pathogenic bacterial loads *in vivo* play an important role in disease (64). Our data showed that disruption of the *hp1330* gene led to a clear decrease of the blood bacterial load during SS2 infection. Thus, HP1330 may contribute to SS2 virulence not only through its potent pro-inflammatory activity but also by influencing the blood bacterial load of SS2. Since the loss of individual PGN hydrolase factors usually has little effect on growth and division (65), we speculate that the PGN hydrolysis of HP1330 may be not responsible for the reduced bacterial load of *Δhp1330* mutant in blood. A moderate inflammatory response maintains a relative immunological balance between pro- and anti-inflammatory actions, which is advantageous for host defense against and clearance of bacterial infections. However, cytokine overexpression can break this balance and contribute to promote organ injury and exacerbate disease progression (66). Infection with the highly virulent SS2 strain can cause excessive inflammation, while the *Δhp1330* mutant strain showed significantly reduced pro-inflammatory ability. Thus, we speculate that deletion of the *hp1330* gene may help in the complete or partial recovery of inflammatory response functions against bacteria, in turn helping to reduce the blood bacterial load during SS2 infection.

In conclusion, our data demonstrated that HP1330 is a novel virulence-related protein of SS2, which shows potent pro-inflammatory activity and influences the bacterial load *in vivo*. Furthermore, through further analyzing the inflammatory signaling pathways induced by HP1330, we found that TLR2 recognition and ERK 1/2 MAPK activation mediate the pro-inflammatory responses of mouse macrophages to HP1330 exposure. These findings increase our understanding of the excessive inflammation and STSLS caused by SS2.

## Ethics Statement

This study was carried out in accordance with the recommendations of the Guide for the Care and Use of Laboratory Animals Monitoring Committee of Hubei Province, China, and the protocol was approved by the Committee on the Ethics of Animal Experiments at the College of Veterinary Medicine, Huazhong Agricultural University.

## Author Contributions

The experiments were performed mainly by QZ and JH, and some experiments were performed with the assistance of JY, ZX, LL, YS, and XS. QZ and JH analyzed the data. The study was conceived and designed by AZ and MJ. QZ, JH, AZ, and MJ wrote the manuscript.

## Conflict of Interest Statement

The authors declare that the research was conducted in the absence of any commercial or financial relationships that could be construed as a potential conflict of interest.

## Supplementary Material

Figure S1 Detecting the peptidoglycan (PGN) hydrolase activity of HP1330. As a substrate for zymogram analysis, SS2 PGN was uniformly added to two protein gels. Following SDS-PAGE: **(A)** one gel was stained with Coomassie blue to observe the positive control lysozyme, the negative control bovine serum albumin (BSA) protein, and HP1330, and **(B)** another gel was stained with methylene blue to detect the PGN hydrolase activity of lysozyme, BSA, and HP1330.
