# Supplementary figures and images for: HP1330 Contributes to Streptococcus suis Virulence by Inducing Toll-Like Receptor 2- and ERK1/2-Dependent Pro-inflammatory Responses and Influencing In Vivo S. suis Loads
Source: Front Immunol. 2017 Jul 31;8:869. doi: 10.3389/fimmu.2017.00869 (PMC5534446; doi:10.3389/fimmu.2017.00869)

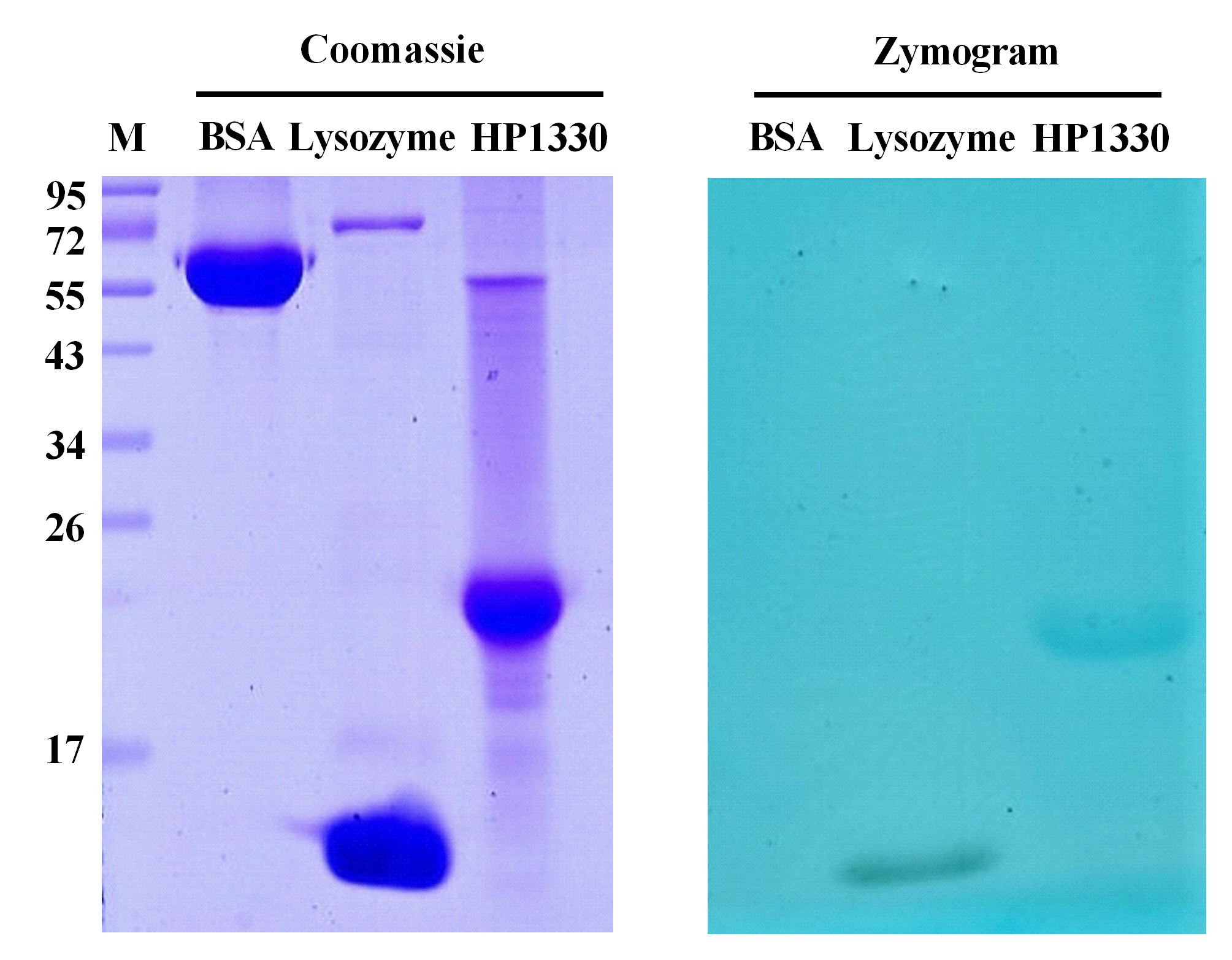

Supplement: Figure S1 — Detecting the peptidoglycan (PGN) hydrolase activity of HP1330. As a substrate for zymogram analysis, SS2 PGN was uniformly added to two protein gels. Following SDS-PAGE: (A) one gel was stained with Coomassie blue to observe the positive control lysozyme, the negative control bovine serum albumin (BSA) protein, and HP1330, and (B) another gel was stained with methylene blue to detect the PGN hydrolase activity of lysozyme, BSA, and HP1330. [file Image_1.JPEG]
